# Comparative histological and immunohistochemical study of ameloblastomas and ameloblastic carcinomas

**DOI:** 10.4317/medoral.21901

**Published:** 2017-04-08

**Authors:** Marisol Martínez-Martínez, Adalberto Mosqueda-Taylor, Román Carlos-Bregni, Fabio-Ramoa Pires, Wilson Delgado-Azañero, Rodrigo Neves-Silva, Beatriz Aldape-Barrios, Oslei Paes-de Almeida

**Affiliations:** 1 Oral Pathology Section, Department of Oral Diagnosis, Piracicaba Dental School, University of Campinas (UNICAMP), Piracicaba, São Paulo, Brazil; 2 Health Care Department, Universidad Autónoma Metropolitana, Xochimilco, Mexico; 3 Pathology Section, Clinical Center of Head and Neck/Hospital Herrera Llerandi, Guatemala City, Guatemala; 4 Department of Oral Pathology, University Federal Fulminense, Rio de Janeiro, Brazil; 5 Department of Oral Pathology, Oral Medicine and Oral Surgery, Faculty of Dentistry, Universidad Peruana Ceyetano Heredia, Lima, Peru; 6 Professor of Oral Pathology, Faculty of Dentistry, Universidad Nacional Autónoma de México, Mexico

## Abstract

**Background:**

This study aimed to compare the histological and immunohistochemical characteristics of ameloblastomas (AM) and ameloblastic carcinomas (AC).

**Material and Methods:**

Fifteen cases of AM and 9 AC were submitted to hematoxilin and eosin (H&E) and immunohistochemical analysis with the following antibodies: cytokeratins 5,7,8,14 and 19, Ki-67, p53, p63 and the cellular adhesion molecules CD138 (Syndecan-1), E-cadherin and β-catenin. The mean score of the expression of Ki-67 and p53 labelling index (LIs) were compared between the groups using the t test. A value of *p*<0.05 was considered to be statistically significant.

**Results:**

All cases were positive for CKs 5, 14 and 19, but negative for CKs 7 and 8. CKs 5 and 19 were positive mainly in the central regions of the ameloblastic islands, while the expression in AC was variable in intensity and localization. CK14 was also variably expressed in both AM and AC. Ki-67 (*P*=.001) and p53 (*P*=.004) immunoexpression was higher in AC. All cases were positive for p63, but values were higher in AC. CD138 was mainly expressed in peripheral cells of AM, with a weak positivity in the central areas, while it was positive in most areas of ACs, except in less differentiated regions, where expression was decreased or lost. E-cadherin and β-catenin were weakly positive in both AM and AC.

**Conclusions:**

These results shows that Ki-67, p53 and p63 expression was higher in AC as compared to AM, suggesting that these markers can be useful when considering diagnosis of malignancy, and perhaps could play a role in malignant transformation of AM. Pattern of expression of CKs 5 and 19 in AC were different to those found in AM, suggesting genetic alterations of these proteins in malignant cells. It was confirmed that CK19 is a good marker for benign odontogenic tumors, such as AM, but it is variably expressed in malignant cases.

** Key words:**Ameloblastoma, ameloblastic carcinoma, immunohistochemistry, odontogenic tumors.

## Introduction

Ameloblastoma (AM) is one of the most common benign odontogenic neoplasms of the jaws, affecting mainly the posterior region of the mandible. It is locally aggressive, with a high rate of recurrence, while ameloblastic carcinoma (AC) is considered its malignant counterpart. Although rare, with less than 120 cases reported in the English literature, AC is the most common malignant odontogenic tumor (1). Microscopically it may show areas reminiscent of ameloblastoma, but with an invasive and less organized pattern, presenting atypia, mitoses and loss of typical ameloblastic morphology (2). Diagnosis of AC sometimes can be difficult, and both, clinical and histopathological features should be considered. The aim of this study was to compare the microscopic and immunohistochemical characteristics of 9 cases of AC and 15 of AM to determine the pattern of expression or differences in location or intensity of expression that may be of assistance in their differentiation.

## Material and Methods

Fifteen cases of solid/multicystic AM and 9 AC were retrieved from the files of 5 Latin American oral pathology diagnostic services (Piracicaba Dental School, Brazil; Head and Neck Clinical Center, Guatemala; Peribact Private Service of Oral Pathology, Mexico; Oral Pathology Laboratory of the Universidad Autonoma Metropolitana Xochimilco, Mexico, and Oral Pathology Laboratory of the Universidad Peruana Cayetano Heredia, Peru). Clinical and radiographic data were retrieved from the patient´s files when available. Histological hematoxylin and eosin (H&E) stained sections of all cases were reviewed and diagnosis confirmed according to the current WHO Histological Classification of Tumors (3). For immunohistochemical analysis the following antibodies were used: cytokeratins 5,7,8,14 and 19, Ki-67, p53, p63 and the cellular adhesion molecules CD138 (Syndecan-1), E-cadherin and β-catenin ( Table 1). For immunohistochemical staining 3µm sections of each case were used. Briefly, after antigen retrieval with EDTA/Tris buffer (pH 9.0) in a microwave oven, endogenous peroxidase activity was blocked with 20% H2O2. After overnight incubation, primary antibodies were detected by secondary antibodies labeled with estrepta-vidin-biotin-peroxidase, and the reaction developed with diaminobenzidine hydrochloride (DAB, Dako). The preparations were lightly counterstained with Carazzi hematoxylin, mounted with Canada balsam and examined by light microscopy. Immunohistochemical results were graded as negative (-) or positive (+). Labeling index (LI) estimated with Ki-67 and p53 nuclear positiveness were evaluated in 5 selected epithelial areas that showed higher cellular density, using a 20X objective, and expressed in percentage using the ScanerScope System (Nuclear V9). The mean score of the expression of Ki-67 and p53 LIs were compared between the groups using the t test. A value of *p*<0.05 was considered to be statistically significant. This work was approved by the Ethics Committee in Research of the Piracicaba Dental School, University of Campinas, Brazil (registration number 120/2013).

**Table 1:**
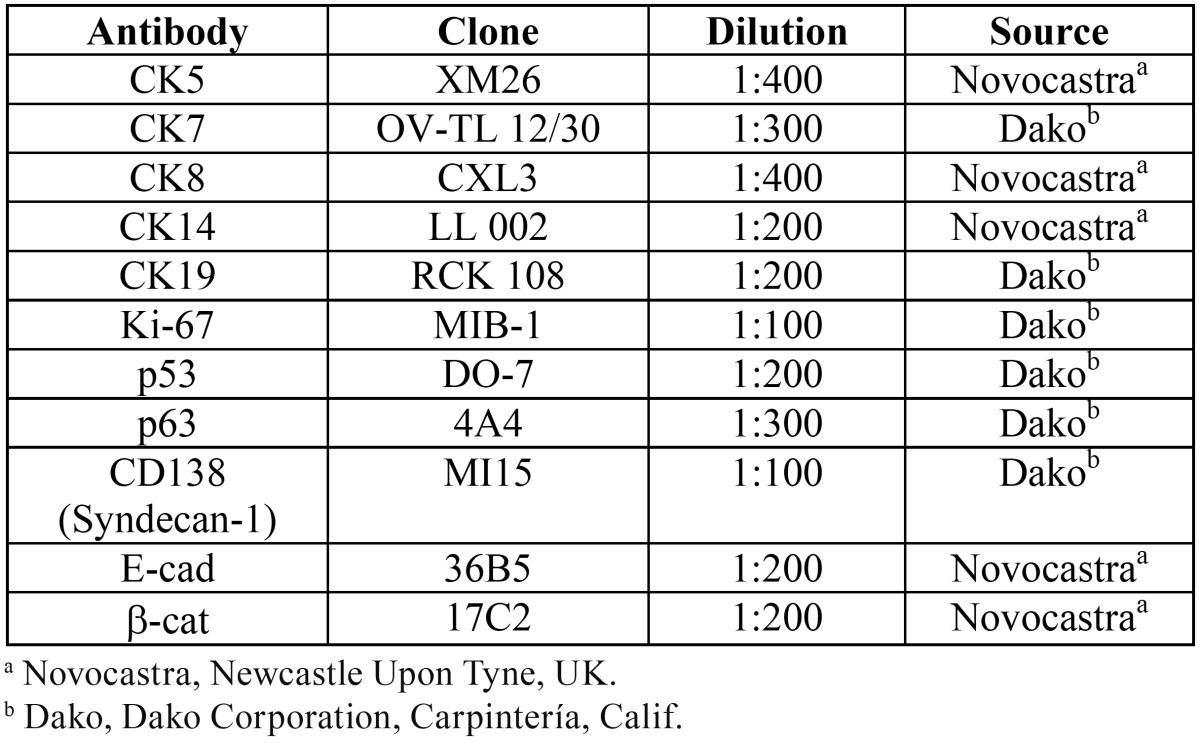
Antibodies used for immunohistochemical evaluation of 15 cases of ameloblastomas and 9 cases of ameloblastic carcinomas.

## Results

Among the 15 cases of AM there were 9 males and 6 females; age ranged from 11-58 yr. (mean age: 33.60 yr.). Nine cases involved the posterior mandible, and histologically all were of the solid type, 12 with predominant plexiform and 3 with follicular patterns. The stroma was variably collagenized, and in 4 cases it was focally myxoid.

**Figure 1:**
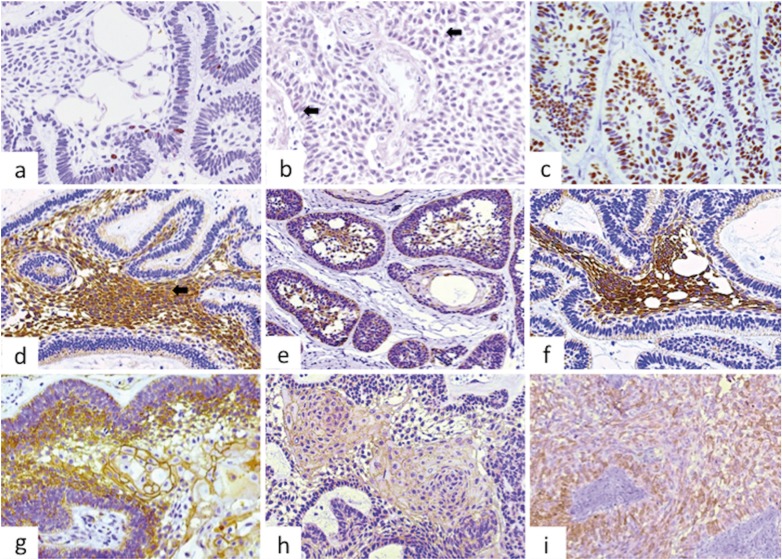
Main immunohistochemical features in ameloblastoma: a. Ameloblastoma showing the nuclei of a few cells of the basal and suprabasal layers positive for Ki-67 (200X), b. Nuclear expression of p53 in AM, positive cells are evident, but the intensity of the staining is weak- arrows (200X), c. p63 immunostaining in ameloblastomas, showing epithelial islands intensely positive mainly in the peripheral cells, but many of the central cells also expressed this marker (200X), d. The central areas of the islands/cords were strongly positive for CK5, while the expression was weaker or negative on the peripheral columnar cells (200X), e. Follicular islands showing stronger expression in the central areas, but the basal layer was also positive. Arrow shows squamous metaplasia with cells weakly positive (200X), f. CK19 immunostaining in ameloblastoma, showing central cells intensely positive, with weak or negative expression in the basal and suprabasal layers (400X), g. Higher magnification of an epithelial island illustrating positiveness of CD138 mainly in the basal and suprabasal layers. Arrow shows squamous metaplasia, with peripheral cells exhibiting strong positivity, with the more central squamous cells loosening expression (400X), h. Central areas of an epithelial ameloblastic sheet showing positivity in cells presenting characteristics of squamous metaplasia (400X), i. Case of AM showing strong positiveness for β-catenin mainly in the peripheral columnar cells, loosing expression in some central cells. This strong staining was not common, as in most of the cases the labeling with β-catenin was weak or practically negative (200X).

Ki-67 was mainly positive in some cells of the basal and suprabasal layers, but only occasional cells in the central areas similar to the stellate reticulum of the enamel organ were marked. The proliferative index varied between 0.35% and 6.89% with a mean of 2.37% (Fig. 1a). The localization and percentage of p53 immunostaining were similar to Ki-67, but it was less intense, with values of positive nuclei between 0.13% and 5.90% and a mean of 1.77% (Fig. 1b). p63 was positive mainly in the columnar cells of the peripheral layer of the islands/cords while in the central areas staining was variable, except for those areas of squamous metaplasia, which were positive in most cases (Fig. 1c).

Regarding CKs expression, CK5 appeared more intensely expressed in the central stellate reticulum-like areas than in the basal columnar cells, and it was positive in islands presenting flattened cells in both the peripheral and central areas (Fig. 1d), while in areas of squamous metaplasia it was variably expressed. CK14 expression was weak and diffuse in most cases, and positiveness was mainly found in the stellate reticulum-like areas (Fig. 1e), but also in the basal layer of cystic areas, and it was weakly positive in some of the most superficial cells. CK19 showed strong expression in the central cells of the cords/islands, but it was weak or negative in the peripheral columnar cells (Fig. 1f). All cases of AM were negative for CK7 and CK8.

Among the adhesion molecules evaluated, CD138 was the most strongly expressed, particularly in the basal and suprabasal layers, while areas of squamous metaplasia were negative or weakly positive, while most central stellate reticulum-like cells were negative (Fig. 1g). E-cadherin expression in AM was not as evident as CD138, and in some cases staining was faint or practically negative. The basal and suprabasal cells exhibited a higher positivity than those located in the central areas. In some cases, groups of cells presenting squamous metaplasia were strongly positive, particularly when cell junctions were evident in H&E (Fig. 1h). β-catenin was the least expressed of the three adhesion markers used, being weakly positive in most cases, except in areas with squamous metaplasia, where expression was more evident (Fig. 1i).

**Figure 2:**
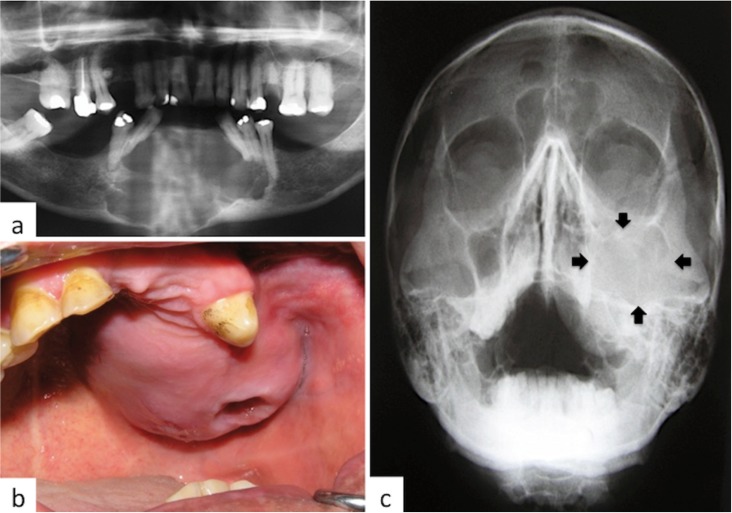
Most cases of ameloblastic carcinoma involved the posterior mandible. This figure illustrates two unusual cases, one involving the anterior mandible and the other the left maxilla, a. Panoramic radiography showing irregular radiolucent lesion involving the anterior mandible causing tooth displacement, mobility and loss, b. AC of left maxilla affecting the alveolar ridge and extending into the palate. The perforation in the mucosa corresponds to a recent tooth extraction, c. Radiograph of the same case showing radiolucent image involving the left maxillary sinus and adjacent areas, including floor of the orbit.

**Figure 3:**
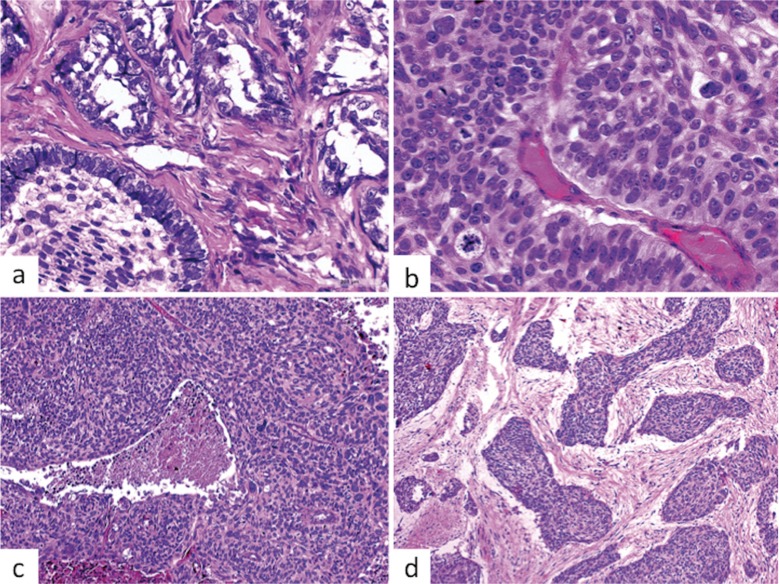
Histological features of ameloblastic carcinoma, a. Epithelial islands with artefactual empty central spaces showing a follicular pattern, resembling an ameloblastic epithelium with columnar peripheral cells. The stroma is highly collagenized (H&E, 400X), b. Sheets of malignant epithelial cells showing a peripheral layer formed by columnar cells, with ameloblastic characteristics, including reversal nuclear polarization. Cellular pleomorphism and atypical mitosis are evident (H&E, 400X), c. Sheets of malignant epithelial cells showing a central area of necrosis (H&E, 200X). d, Islands of well-defined malignant epithelium, with flattened peripheral cells separated by collagenized stroma. The islands are frequently separated from the adjacent stroma, suggesting a poor adherence of epithelium/stroma adherence interface (H&E, 200X).

With respect to the 9 cases of AC, there were 5 males and 4 females, with a mean age of 43.67 years (range: 22-65 years). The mandible was involved in 8 cases, 7 in the posterior region, and only one case occurred in the maxilla (Fig. 2). All cases were solid, with only one showing cystic areas. Most cases were composed by sheets of cells arranged in a plexiform pattern, but in 2 cases a follicular pattern predominated. The malignant epithelial cells showed pleomorphism and mitoses, and in six cases there were evidence of focal areas of necrosis. Some islands showed central areas similar to the stellate reticulum of the enamel organ, and most cases contained areas reminiscent of benign ameloblastoma but these were exceeded by the malignant component. The stroma usually was scarce and collagenized (Fig. 3).

**Figure 4:**
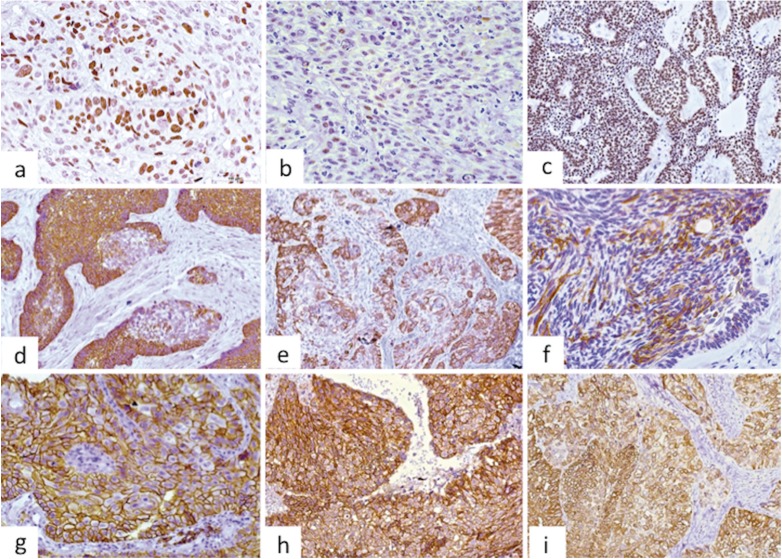
Main immunohistochemical features in ameloblastic carcinoma: a. Epithelial cells showing strong and high expression of Ki-67 in most of the cells (200X), b. Nuclear expression of p53 in AC, positive cells are evident, but the intensity of the staining is weak (200X), c. p63 immunostaining in ameloblastic carcinoma, with most of the nuclei strongly positive (200X), d. Strong and homogenous expression of CK5 in AC, both in the central and peripheral cells of the epithelial islands, with loss of expression in focal areas (200X), e. Neoplastic islands illustrating strong expression for CK14 in most cells, but loss of staining or weak expression in focal regions (200X), f. CK19 staining in ameloblastic carcinoma, showing strongly positive cells permeated by negative cells in both peripheral and central areas (200X), g. epithelial malignant sheet showing most cells with strong positivity for CD138 (400X), h. Case of AC strongly expressing E-cadherin in most of the cells; nevertheless, expression was variable in most cases, and some were negative (200X), i. Case of AC showing strong expression of β-catenin. In most cases it was weakly positive or negative (200X).

Ki-67 proliferative index in AC ranged from 11.87% to 53.29% (mean: 23.46%), with positiveness in both peripheral and central cells (Fig. 4a). Regions similar to benign ameloblastoma exhibited a lower expression of Ki-67, but these were not included for quantitation. The distribution of cells positive for p53 was similar as above described for Ki-67, but the nuclei were much less intensely stained, with percentages of positive cells varying from 11.57% to 80.88% (mean: 36.01%) (Fig. 4b). p63 expression was strongly positive in most cases and in most cells (Fig. 4c).

Expression of CK5 in AC was variable, as in 7 cases it was strongly positive either at the periphery or within the central epithelial cells, while two cases showed total or partial loss of CK5 in most areas, being positive only in a few more differentiated islands still showing ameloblastic characteristics (Fig. 4d). Expression of CK14 was also variable in intensity and localization, with 5 cases presenting positive expression in most of the cells, while in 4 it was either negative or focally positive (Fig. 4e). CK19 expression was not uniform, showing areas strongly positive mainly in a few islands with ameloblastic characteristics (Fig. 4f). Two cases of AC were focally positive for CK7 and CK8.

CD138 was strongly positive in most cases, except in very undifferentiated areas where its expression was lost or it was weakly positive (Fig. 4g). Expression of E-cadherin was variable both in intensity and localization (Fig. 4h). β-catenin expression was weak or negative in all but one case (Fig. 4i). Main immunoprofile and comparison between ameloblastomas and ameloblastic carcinoma are shown in  Table 2.

**Table 2:**
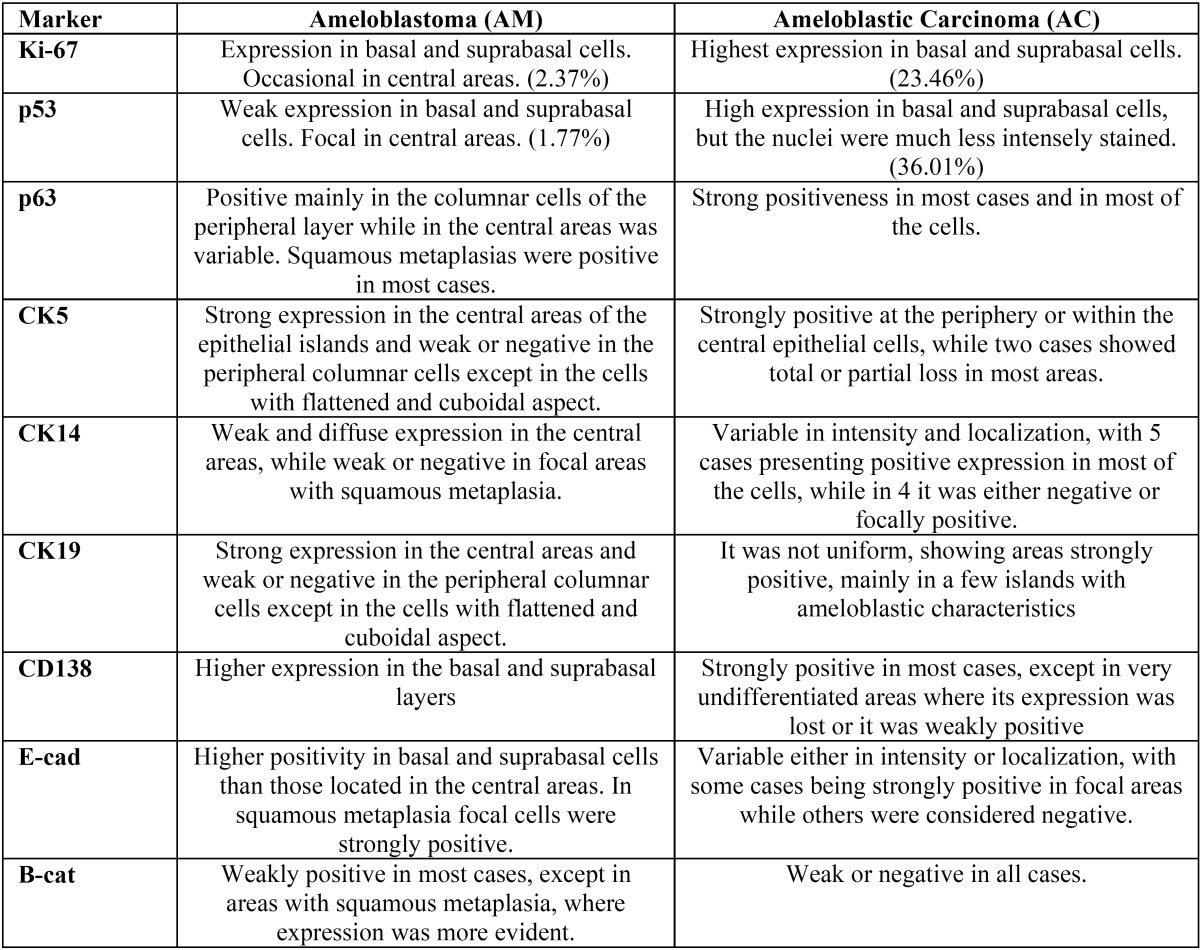
Comparison of the main immunohistochemical features of 15 cases of ameloblastomas and 9 cases of ameloblastic carcinomas observed in this series.

## Discussion

AMs are locally aggressive benign tumors with a relatively high risk of recurrence and potential to malignant transformation into AC. Up to date, there are around 120 cases of AC reported in the English literature, some of which seem to have developed from AM (4-5). Radiographically, AM may appear either as uni or multilocular osteolytic lesions that frequently produce cortical expansion. If paresthesia, pain, irregular borders and invasion of adjacent structures are present, then a malignant lesion should be suspected (2,6).

In contrast to AM, ACs usually present microscopic evidence of malignancy, but confirmation of the diagnosis of a secondary type of AC (those developed from a pre-existent AM) need to be supported by a history of persistent or recurrent AM, or the presence of benign residual areas of AM; eventually, differential diagnosis between these two lesions can be difficult, particularly in incisional biopsies, and therefore other types of intra-osseous carcinomas of the jaws should be considered. Immunohistochemistry is not usually employed to assist diagnosis and classification of odontogenic tumors, but some immunomarkers may be useful for a better understanding of their origin and possible biological behavior, and therefore the immunohistochemical profile of these lesions may sometimes be helpful to reach a correct diagnosis in doubtful situations. The present series confirms that Ki-67 proliferative index (*P*=.001) and p53 expression (*P*=.004) are higher in ACs than in AMs. These results confirm that Ki-67 immunoexpression is a useful tool to distinguish among benign and malignant tumors, as it has been demonstrated in the malignant areas of other tumors, such as carcinoma ex-PA with scant areas of malignancy that are not clearly evident under H&E stained sections (7-8). The higher expression of p53 found in ACs as compared to AMs suggest that this protein may play a role in malignant transformation, as has been also suggested by other authors (9-11). However, data regarding p53 expression in ACs varies in different studies; for example, studies performed by Yoon *et al.*, (2); Karakida *et al.*, (12); Matsuzaki *et al.* (10), and Nobusawa *et al.*, (13) described p53 overexpression in almost all tumor cells of the carcinoma component, while it was negative in the benign ameloblastic areas; however, Abiko *et al.* (14) found that both benign and malignant areas of one case of AC were negative for p53 by immunohistochemistry. In our opinion the information obtained in a single case is not enough to compare with our findings, and with those found by the other authors that based their studies in series of cases that have followed a similar sample processing and analysis, with less probabilities of misdiagnosis and technical errors.

It is well known that CKs 14 and 19 are regularly expressed in the epithelium of the dental germ, where CK14 is found in the diverse components during all stages of tooth development, including the dental lamina, the inner enamel epithelium and in almost all cells of the enamel organ, while CK19 is mainly positive in the later stages of development, during terminal differentiation of ameloblasts (15,16). We found a similar pattern of expression of these proteins in AM, but it was different to the one observed in AC since in the malignant tumor the expression was irregular in intensity and location for both CKs. CKs 5, which was less expressed in AC than in AM and CK14 seem to be the major CKs expressed by epithelium in the human enamel organ, marking the end of secretion of amelogenin (17).

CK8 is found in embrionary and several simple epithelia, as well as in the human enamel organ, but Crivellini *et al.*, concluded that CK8 was absent in odontogenic epithelium, as it was observed in most cases in our study.

CK19 expression has been demonstrated in most odontogenic cysts and benign tumors, and it has been considered a good marker for benign odontogenic lesions (14). Nevertheless it is also expressed in AC and therefore has no diagnostic utility to differentiate among these entities (18,19). In the present study, CK19 expression in AC was variable, being usually maintained in areas with ameloblastic characteristics.

p63 is expressed in normal epithelium and it is often overexpressed in carcinomas, including oral squamous cell carcinoma (20). It is also overexpressed in odontogenic carcinomas, such as AC, primary intraosseous squamous cell carcinoma and clear cell carcinoma as compared to benign odontogenic lesions, suggesting that p63 may have a role in tumorigenesis of odontogenic lesions (21). In the present study, p63 was mainly expressed in the basal and suprabasal cells of AM, in a pattern previously observed in odontogenic keratocyst, suggesting that this protein is a good marker of more immature keratinocytes (22). Different from AM, p63 was overexpressed in all our cases of AC, labeling most of the cells, in a similar way as it occur in other types of carcinomas, indicating lack of cell differentiation and a higher potential for growth, two common characteristics of malignancies (23).

Cell adhesion molecules and their interactions can be altered in tumors, particularly in malignancies, favoring progression, invasion, recurrence and metastasis (5). In normal conditions, E-cadherin is moderately expressed in odontogenic epithelium during the bell stage, especially in the stellate reticulum of the enamel organ, and also in the polyhedral cells of ameloblastomas (24). β-catenin is a multifunctional molecule that plays a role in cell-cell adhesion mediated by classic cadherins as well as in Wnt-signaling (25). CD138 is a cell surface proteoglycan present in normal epithelial cells, whose expression seems to decrease in ameloblastomas, as compared to epithelial cells of normal enamel organ, having also an inverse relation with cell proliferation index, aggressiveness and recurrence (26-28). There are some studies that have demonstrated positive expression of CD138 in AM, both in the columnar and the polyhedral cells of the stellate reticulum (29), and nuclear expression of E-cadherin and β-catenin have been reported in cases of solid AM and odontogenic carcinomas (25). In this study, CD138 was the most strongly expressed adhesion molecule, both in AM and AC, while E-cadherin and β-catenin expression was faint in most of the cases. These last two markers were intensely positive only in areas of squamous metaplasia of AMs, particularly when desmosomes were evident in H&E stained preparations. CD138 expression was faint or negative only in the central areas of AMs islands where cells were loosely adhered, leaving spaces between them. CD138 was positive in most areas of AC, except in the less differentiated regions, where expression was decreased or lost. However, our data does not allow us to confirm that there is a higher loss of these molecules in AC as compared to AM, and therefore more studies are necessary to determine if loss of expression of these proteins can be relevant to support malignant transformation or higher aggressiveness in these neoplasms. As mentioned above, expressions of E-cadherin and β-catenin were weak in AMs and ACs, difficulting analysis about their possible role in these neoplasms.

In summary, the results of this work confirmed that patients with AC were about a decade older than those with AM. Ki-67 and p53 immunoexpression was higher in AC than in AM, supporting the idea that they can be good markers of malignant transformation. p63 was also more frequently expressed in AC, suggesting loss of maturation of the malignant epithelial cells. The pattern of expression of CKs 5 and 19 is altered in AC in relation to AM, but this does not seem to be related to malignant transformation. Finally, we could not find evident loss of the adhesion molecules CD138, E-cadherin and β-catenin in AC in relation to AM, but as ACs are very rare lesions, we suggest that new series of cases need to be studied for a better understanding of its clinical and biological aspects.
